# Simplified Early Predictors of Severe Acute Pancreatitis: A Prospective Study

**DOI:** 10.4021/gr2010.02.172w

**Published:** 2010-01-20

**Authors:** Maha Mohammed Maher, Basma Abdel Moneim Dessouky

**Affiliations:** a Internal Medicine Department, Mansoura University, Mansoura, Egypt; b Radiodiagnosis Department, Menoufeya University, Menoufeya, Egypt

**Keywords:** Severe acute pancreatitis, CT Balthazar grading system

## Abstract

**Background:**

To propose simple tests for the prediction of severe acute pancreatitis (SAP), which are accurate and could be performed at emergency departments and outpatient clinics.

**Methods:**

A prospective study was performed on 149 patients admitted with acute pancreatitis. Body mass index (BMI), plain chest radiograph, blood biochemical data were obtained at the time of admission; white cell, lymphocyte and platelet counts, hematocrit level, prothrombin time, PaO_2_, creatinine, calcium, blood sugar, total protein, aspartate aminotransferase, total bilirubin, amylase, lipase and C-reaction protein were determined. Patients were graded into severe and mild acute pancreatitis based on CT Balthazar grading system.

**Results:**

Twenty-seven patients were diagnosed to have SAP and 122 patients considered mild acute pancreatitis. Comparing parameters between both groups; significant factors (P < 0.05) were blood sugar level, haematocrit level, BMI and presence of pleural effusion in chest X-ray. The hematocrit at admission and at approximately 24 hours was significantly higher among patients with SAP. Twenty-two of 27 cases of severe disease and only 10 of 122 cases of mild acute pancreatitis diagnosed to have pleural effusion (P < 0.001).

**Conclusion:**

BMI, blood glucose ≥ 190 mg/dL, hematocrit level ≥ 43 % and pleural effusion detected by plain chest radiograph are simple tests and provide significant predictive power for clinical decision-making.

## Introduction

Acute pancreatitis (AP) has been defined as an “acute inflammatory process of the pancreas with variable involvement of other regional tissues or remote organ systems” [1]. Despite recent advances of diagnosis and treatment modalities, mortality rate in severe acute pancreatitis (SAP) is still high [2]. Over 25% of cases involving AP are severe, with mortality as high as 10% [3, 4]

The early and accurate staging of the severity and the assessment of prognosis of the disease are of great clinical significance so that the urgent and most suitable therapy can be provided for the AP patient. In recent years, several clinical and imaging systems for scoring the severity and prognosis of AP have been developed, including Ranson's criteria, acute physiology and chronic health evaluation (APACHE II) and CT criteria [5] and so on. Most of these tests either are difficult to remember, take greater than 48 hours to enable full severity stratification, or rely on diagnostic testing that is not widely available [6]

The search for a tool that can improve on the clinical assessment has been ongoing for decades. Ideally, such a tool should be simple, inexpensive, noninvasive, easily applicable, and reproducible and have the statistical power to arrive to a valid conclusion accurately [7]. In recent decades, several authors have indicated that CT can help in determining the prognosis of AP; the CT findings in these works correlated with clinical course, complications, and mortality in patients with AP [8].

This study aimed to propose simple prognostic tests for the prediction of the of SAP, which are accurate and could be performed at most emergency departments and outpatient clinics.

## Patients and Methods

### Patients

A prospective study was conducted on 149 patients with AP admitted king Fahd Hospital Hofuf (a major tertiary hospital in eastern region in Saudi Arabia ) from January 2005 to June 2008; 82 males and 67 females; age, 42.4 ± 1.9 years. AP was defined by the presence of a clinical illness compatible with this diagnosis and the absence of another diagnosis that would explain the symptoms associated with the serum amylase or lipase concentration at least three times the upper limit of normal. Patients with AP and onset of pain less than 72 h before admission were considered for the study. At the time of admission, the diagnosis and evaluation of the severity of AP were made according to the CT criteria.

Gender, age, weight and height on hospital admission, AP etiology; alcohol was considered as the cause of AP when a history of heavy ethanol intake before the episode was documented and other etiologies were discarded. Diagnosis of biliary AP was sustained by ultrasonographic findings of gallstones or bile duct dilatation and/or serum liver chemistry compatible with obstructive jaundice, without other obvious cause of the attack.

Criteria of exclusion to the study were the following; age less than 18 years, diabetes mellitus, history of allergy to intravenous contrast medium, history of pancreatic carcinoma or chronic pancreatitis, pregnancy, the presence of a severe debilitating illness such as neoplasm, acquired immunodeficiency syndrome, or collagen vascular disease or illnesses that could compound the interpretation of the investigations such as known anemia, presence of pleural effusion on chest radiograph preceding the development of AP, a comorbid medical condition that could lead to effusion (such as congestive heart failure), and inability of patients to cooperate.

### Study design

Plain chest radiograph, blood biochemical data; white blood cell count, lymphocyte count, platelet count, hematocrit level, prothrombin time, PaO_2_ level, creatinine level, BUN level, calcium level, blood sugar level, total protein level, aspartate and alanine aminotransferase levels, total bilirubin level, amylase level, lipase level and CRP level were analyzed.

Samples were obtained at the time of admission (within the initial 72 hours after the onset of disease), time interval between onset and admission was 48 ± 3 hours. All mentioned parameters were repeated every 24 hours after patient admission.

### Contrast enhanced CT

On admission Contrast-enhanced CT was performed to evaluate the extent of inflammation and necrosis in all patients with AP. The following items of imaging were analyzed: (1) size of the pancreas, (2) degree of uptake of contrast medium by pancreatic parenchyma, (3) extent of necrosis of pancreatic parenchyma, (4) extent of pancreatic inflammation, (5) peripancreatic fluid collections and (6) presence of stones in the gallbladder and common bile duct.

The staging of severity on CT was based on Balthazar grading system (A-E) [9], stage A is normal morphology of the pancreas, stage B is characterized by diffuse or localized enlargement of the pancreas with heterogeneous attenuation of pancreatic parenchyma, stage C is peripancreatic spread of inflammation to retroperitoneal space, stage D is characterized by single fluid collection and stage E by multiple fluid collections.

If pancreatic necrosis was absent on admission, CE-CT was performed again 2 days after admission, and the diagnosis was confirmed. Afterward, CE-CT was performed once each week. If pancreatic necrosis was present on admission afterward, CE-CT was performed once each week.

All AP patients were graded into two types: mild and severe AP. The mild AP was consistent with the following scores including stage A to C of Balthazar's CT scan score. While the severe AP was consistent with stage D and E of Balthazar's CT scan score.

From data that were obtained at the time of admission, the factors of statistically significant difference that were observed in the 2 groups were surveyed.

Analyzed parameters were plain chest radiograph, blood biochemical data; white blood cell count, lymphocyte count, platelet count, hematocrit, prothrombin time, PaO_2_, creatinine, BUN, calcium, blood sugar, total protein, aspartate and alanine aminotransferase, total bilirubin, amylase, lipase, and C-reaction protein (CRP).

Significant prognostic factors were then analyzed by receiver operator characteristic (ROC) curves [10]. The area under the curve (AUC) was evaluated, and the optimum cutoff level for each factor was determined, multivariate analysis was carried out among the significant prognostic factors to determine independent variables that were associated with SAP and patients who developed organ failure.

### Statistical Analyses

Results are expressed as mean ± SD. The Mann-Whitney U test was used to evaluate differences between the 2 groups AUC and optimum cutoff level of each factor was determined by analyses of ROC curves. The sensitivity, specificity, positive and negative predictive values for each of the 4 single criteria, CT gradings were calculated. Factors found to be significant on univariate analysis were entered into a multivariate logistic regression analysis. P < 0.05 was considered statistically significant. All statistical comparisons were performed using SPSS Version 12.0.

## Results

There was a total of 149 patients with AP who met the study inclusion criteria; of whom 67 (45%) were women and 82 (55%) were men; age 42.4 ± 1.9 years. The etiology of AP was biliary in 77 patients (51.7%) and nonbiliary in 72 patients (48.3%). All patients underwent contrast-enhanced CT scan.

Using the results of enhanced CT performed at admission and after 48 hours, a total number of 27 (18.1%) patients (15 males and 12 females) with a mean age of 47 ± 2.1 years was diagnosed to have SAP (group I), grade D, 12 patients (8.1%); grade E, 15 patients (10.1%). The remaining 122 (81.9%) patients considered mild AP (group II) with a mean age of 43 ± 2 years, grade A, 58 patients (38.9%); grade B, 26 patients (17.4%); grade C, 38 patients (25.5% ). All complications occurred in the severe group, mainly in the patients with grade E disease, with statistically significant differences for both morbidity (p < 0.002) and mortality (P < 0.05) as compared with patients with mild disease. Demographic data of both groups are represented in Table 1.

**Table 1:** Demographic Data of Patients With Severe and Mild Acute Pancreatitis

	Severe Pancreatitis (n= 27)	Mild Pancreatitis (n=122)	P Value
Age (years)	47 ± 2.1	43 ± 2	0.21
Sex (male/female)	15/12	67/55	0.76
Etiology of AP			
Gall stones	15	62	0.58
Non gall stones	12	60	0.96
Time of hospitalization (days)	25 (12-38)	7 (5-9)	0.03
BMI (kg/m^2^)	30.9 ( 27.9 - 34.2)	25.4 (23.4 - 28.1)	0.007
Patients with EOF			
Respiratory	11	0	
Renal	3	0	
Shock	1	0	
Total	15	0	0.002

EOF: End Organ Failure

Comparing parameters between group I and group II, significant factors (P < 0.05) were blood sugar, haematocrit, BMI and presence of pleural effusion in chest X- ray (Table 2). These 4 significant prognostic factors were then analyzed by ROC curves, and AUC was evaluated according to their usefulness. Multivariate analysis among these significant factors revealed that the independent predictable factors for prognosis were blood glucose (P < 0.01) and hematocrit (*P* = 0.01). The optimum cutoff level of blood sugar was 190 mg/dL and hematocrit was 43%.

**Table 2:** Significant Prognostic Factors Between Severe and Mild Pancreatitis Groups by Univariate Analysis

	Group I (n = 27)	Group II (n = 122)	P Value
Age (years)	53 ± 9	49 ± 2	0.21
BMI			
25 - 30 kg/m^2^	11	12	0.03
≥ 30 kg/m^2^	14	6	0.02
Creatinine (mg/dL)	1.9 ± 0.6	1.1 ± 0.3	0.11
BUN (mg/dL)	33 ± 4	27 ± 2	0.55
CRP (mg/dL)	20 ± 2	18 ± 1	0.07
WBCs (cells / mm^3^)	9 ± 7.5	7 ± 3.1	0.82
Hematocrit (%)	46.1 ± 5.9	41.8 ± 4.2	0.001
Blood sugar (mg/dL)	273 ± 31	149 ± 3	0.01
Calcium (mg/dL)	6.0 ± 0.3	7.4 ± 0.1	0.67
Total bilirubin level (mg/dL)	2.1 ± 0.4	1.7 ± 0.2	0.09
AST (IU/L)	128 ± 65	112 ± 16	0.07
Amylase (U/L)	731 ± 176	638 ± 81	0.11
Lipase (U/L)	903 ± 96	711 ± 47	0.34
Chest X-ray (no.)	22	10	< 0.001

BMI: Body Mass Index; BUN: Blood Urea Nitrogen; CRP: C reactive protein; WBC: White Blood Cell; AST: Aspartate aminotransferase

BMI was higher in SAP patients (group I) compared to mild AP (group II) (median, 30.9 kg/m^2^, IQR, 27.9-34.2 kg/m^2^ vs mean 25.4 kg/m^2^, IQR, 23.4-28.1 kg/m^2^; P = 0.007). The number of obese patients in group I (BMI ≥ 30 kg/m^2^) was 14 (51.9%) and 6 (4.9%) in group II (P = 0.02).

The sensitivity of BMI as a marker for SAP was 92.3% and a specificity of 85.2% (positive predictive value, 58.1%; negative predictive value, 98.1%).

Plasma glucose was significantly higher in group I vs group II in patients with normal body weight, overweight patients (BMI, 25 - 30 kg/m^2^), and obese patients (BMI, ≥ 30 kg/m^2^) (P = 0.05, 0.001, 0.002 respectively). BMI and plasma glucose level had linear correlation in group I patients (r = 0.451, P = 0.001), but no correlation was seen in group II patients (r = 0.084, P = 0.88). Blood glucose had a sensitivity of 86.1% and a specificity of 90.6% (positive predictive value, 69.3%; negative predictive value, 95.8%). A serum glucose level of (190 mg/dL) or higher, was the best single, simple predictor of severe pancreatitis in non diabetic patients.

There was no significant difference in age, gender, interval of time between the onset of pain and admission, or interval of time between the first and second hematocrit measurements among group I and group II patients ( P = 0.21, 0.23, 0.98 and 0.34, respectively). Hematocrit at admission and at approximately 24 hours was significantly higher among patients with SAP. The change in hematocrit between 24 hours and admission was not significantly different Table 3.

**Table 3:** Time Intervals and Hematocrit Values in Severe Versus Mild Pancreatitis

	Group I (n = 27)	Group II (n = 122)	P Value
Interval of time between onset of pain and admission (h)	18 (6, 72)	20 (7, 84)	0.98
Interval of time between first and second hematocrit measurements (h)	24 (20, 28)	24 (18, 25)	0.34
Hematocrit at admission (%)	46.1 (38.4 - 51.4)	41.8 (31- 47.1)	< 0.001
Hematocrit after 24 hours (%)	43.9 (31.4 - 53.9)	37.8 (26 - 46.6)	< 0.001

The sensitivity of hematocrit as a marker for SAP was 76% at admission and 95% at 24 hours; specificity was 81% and 71%, respectively. The positive predictive value was 70% at admission and 63% at 24 hours; the negative predictive values were 81% and 92%, respectively (Table 4).

**Table 4:** Hematocrit as a Marker for Severe Acute Pancreatitis

Severe Acute Pancreatitis	Sensitivity	Specificity	PPV	NPV
Hematocrit criteria				
At admission	76%	81%	70%	81%
	(43%, 89%)	(64%, 93%)	(44%, 87%)	(73%, 92%)
At 24 hours	95%	71%	63%	92%
	(78%, 100%)	(55%, 84%)	(43%, 80%)	(77%, 96%)

PPV: positive predictive value; NPV: negative predictive value

Chest radiographs were performed in all cases: 22 of 27 cases (81.5%) of severe disease (group I); only two patients pleural effusion was detected on admission, and only 10 of 122 cases (8.2%) of mild AP (group II) diagnosed to have pleural effusion, this difference was statistically highly significant (P < 0.001). Pleural effusion had a sensitivity of 81.4% and a specificity of 91.8% (positive predictive value, 68.8%; negative predictive value, 95.7%). However, there was no significant correlation between the etiology of pancreatitis and the incidence of pleural effusion.

Effusions were bilateral in (68.8%), left-sided in (25%), and right-sided in (6.3%). The distribution of effusions was similar in mild and severe pancreatitis: bilateral effusions comprised 72.7% of all effusions in severe disease and 60% of all effusions in mild disease. Results of pleural effusion distribution are reported in Table 5.

**Table 5:** Distribution of Pleural Effusion in Severe Versus Mild Acute Pancreatitis

	SAP with effusion (N = 22)	Mild AP with effusion (N = 10)	Total (N = 32)
Left-sided	5	3	8 (25%)
Right sided	1	1	2 (6.3%)
Bilateral	16	6	22 (68.8%)

We evaluated whether pleural effusion was identified before or after recognition of severe disease by CT grading; in the 22 cases of severe pancreatitis and pleural effusion diagnosed after admission, the median day of detection of effusion was hospital day 4 (range, 2-8). We compared this day with the day of detection of severity CT scan; of the 22 patients, in 12 (54.5%) detection of effusion preceded identification of pancreatic necrosis by CT. Therefore, 12 patients with severe pancreatitis did effusion predict severity in the absence of other readily identifiable predictors of severity, namely, necrosis detected by CT or organ dysfunction.

## Discussion

The development of prognostic rules for SAP is believed to be important to the early management of SAP. Animal and human studies have shown that acute events that lead to the development of severe complications of AP develop within 72 hours of the onset of symptoms [11-13]. Several biologic markers and clinical scoring systems have been used to predict the course of SAP. However, conventional multiple-factor scoring systems are complicated and hard to use. Therefore, there is a need for a simple scoring system that can be applied easily with high accuracy and sensitivity on admission [14].

In our study, we selected the most simple parameters including patient criteria, laboratory tests and imaging techniques that can be easily applied in emergency room.

The definitions of severe pancreatitis used in the various studies have not been clear or consistent. Ranson et al [15] defined it as pancreatitis associated with the development of "life-threatening complications or requiring more than 7 days of ICU monitoring." The Glasgow group defined severe pancreatitis as pancreatitis that "failed to settle" [16]. In another study that compared the 3 versions of the Imrie (Glasgow) scoring system, severe pancreatitis was defined as the "development of a major complication requiring more than 20 days hospitalization" [17]. From these examples, it is clear that the criteria for defining severe pancreatitis differ from study to another.

Imaging methods have contributed significantly to the staging of severity and prognostic assessment of AP which determine the appropriate management. The classification by Balthazar et al [9] is the reference grading system which has been validated and is used internationally. In our study, diagnosis of severity and patient classification into mild and SAP groups were based on Balthazar grading system.

From analyzed parameters between the two groups, statistical significant differences were found in only four factors; blood sugar, haematocrit, BMI and presence of pleural effusion in chest X-ray.

BMI was higher in SAP patients (group I) compared to mild AP (group II) (P = 0.007), the number of obese patients in group I (BMI ≥ 30 kg/m^2^) was 14 (51.9%) and 6 (4.9%) in group II (P = 0.02). Obesity has been associated with severe AP in several reports [18-20]. Jorge et al found that 27% of the obese patients had a bad prognostic score on admission as compared with only 11% of the non obese subjects [21].

We excluded from the study patients with history of diabetes, however, plasma glucose was significantly higher in group I compared to group II in patients with normal body weight, overweight patients (BMI, 25-30 kg/m^2^), and obese patients (BMI, ≥ 30 kg/m^2^) (P = 0.05, 0.001, 0.002, respectively). All patients who developed organ failure had necrotizing AP, and thus, pancreatic β-cell mass could be reduced, which results in hyperglycemia [22]. It has also been shown that in an experimental setting, AP results in impaired insulin secretion in the pancreas even in the absence of necrosis [23]. Insulin resistance, an inability of the insulin to increase glucose uptake and use [24], may also be implicated in hyperglycemia in AP. Recent study [25] revealed that hyperglycemia was more remarkable in obese patients, same findings in our study population.

Our study showed that hematocrit at admission and at approximately 24 hours was significantly higher among patients with SAP. The change in hematocrit between 24 hours and admission was not significantly different, study of Brown et al supported this result [26].

The relationship of hemoconcentration to the development of pancreatic necrosis has been studied extensively in the experimental animal. These studies have shown that hemoconcentration early in AP contributes significantly to the impairment of the pancreatic microcirculation and to the progression to pancreatic necrosis [27, 28].

Previous reports have noted a correlation between pleural effusion and severity in AP, as evidenced by necrosis and mortality [29, 30]. Another study linked pleural effusion detected by ultrasonography with severe pancreatitis in accordance with criteria outlined by the Atlanta Symposium [31]. However, none of these studies evaluated pleural effusion detected by chest radiography as a predictor of severity using CT. Data from this study demonstrates a correlation between pleural effusion on chest radiograph and severity in accordance with CT findings. The findings of this study confirmed the link between pleural effusion and severity of AP.

However, our study also shows that pleural effusion is rarely an early, independent predictor of severity; only 2 of 27 patients with severe pancreatitis had a pleural effusion on admission. We found no statistical significant relationship between severity of AP and anatomic distribution of pleural effusions. In both mild and severe disease, the majority of effusions were bilateral.

Our study had many strength points, most previous studies were retrospective while our prospective one stressed on highly selected patient criteria. Our patient population was not diabetics which, up to our knowledge, was not included in previous studies of blood glucose in AP. Also, detection of pleural effusion by chest radiograph which considered to be simple and easily applicable than ultrasonography.

To summarize, BMI was significantly higher in SAP patients, because BMI was associated with admission hyperglycemia, we would recommend the use of admission glucose in addition to BMI in prognostic evaluation of patients with AP. A serum glucose level of (190 mg/dL) or higher, was the best single, simple predictor of severe pancreatitis in non diabetic patients. Patients with an admission hematocrit ≥ 43% which does not decrease at approximately 24 hours are at high risk for the development of SAP. Pleural effusion can predict severity in the absence of other readily identifiable predictors of severity, detected by CT or organ dysfunction.

Patients with these criteria should be admitted urgently to an intensive care unit for vigorous fluid resuscitation, supportive care. Additional studies are required to be applied in different localities and more patients number for possible application of simple scoring system including these four parameters. Patients who do not exhibit these criteria have a very low likelihood of developing these complications and in general do not require treatment in an intensive care unit.

In conclusion, BMI, blood glucose, hematocrit level and pleural effusion detected by plain chest radiograph are simple tests and provide significant predictive power for clinical decision-making. Application of these factors on admission may be helpful for the improvement of treatment outcome in SAP.
